# Acute Disseminated Encephalomyelitis Associated With Chronic Cannabis Abuse

**DOI:** 10.7759/cureus.22551

**Published:** 2022-02-23

**Authors:** Tugce Akcan, Milan Gajera, Javad Najjar Mojarrab, Abuzaid Medani

**Affiliations:** 1 Internal Medicine, Marshfield Clinic Health System, Marshfield, USA; 2 Hospital Medicine, Marshfield Medical Center, Marshfield, USA

**Keywords:** acute disseminated encephalomyelitis, central nervous system, cannabis abuse, neuroimaging, encephalomyelitis

## Abstract

Acute disseminated encephalomyelitis (ADEM) is a rare monophasic immune-mediated inflammatory disorder characterized by multifocal demyelinating lesions of the central nervous system. Clinically, it is distinguished by a variety of acute neurological deficits, including varying degrees of mental state changes and white matter abnormalities detected by magnetic resonance imaging (MRI). We present a challenging case of a young woman who developed ADEM as a result of chronic cannabis abuse. This, to the best of our knowledge, is the second case report of ADEM linked to cannabis abuse.

## Introduction

Acute disseminated encephalomyelitis (ADEM) is an autoimmune demyelinating disease of the central nervous system (CNS) [1]. ADEM is also known as post-infectious encephalomyelitis because it has been linked to upper respiratory, gastrointestinal, viral, or bacterial infections [2]. We present the case of a 35-year-old woman who presented to the emergency room with neurological symptoms consistent with a CNS disorder. The results of laboratory and cerebrospinal fluid (CSF) analysis, neuroimaging, and histopathological evaluation of a brain biopsy were all highly suggestive of ADEM likely triggered by chronic cannabinoid abuse.

## Case presentation

A 35-year-old woman with a history of polysubstance abuse, posttraumatic stress disorder, and bipolar disorder was taken to the emergency department after her father discovered her crawling on the floor. Her mental status was last known to be normal 10 to 14 days prior to this presentation. The patient's personal medical history was unavailable due to her aphasia. Her parents stated that she had not experienced any recent signs or symptoms of an upper respiratory infection. She had not had any vaccinations in a long time. On physical examination, there was no sign of trauma or assault. Except for an increased blood pressure of 151/80 mmHg, her vitals were unremarkable. The patient was awake, had a Glasgow coma scale (GCS) of 10, and was able to maintain her airway. She had expressive aphasia, right-sided hemiparesis, hypertonia, and hyperreflexia, with a preference for the left gaze.

Except for an elevated white blood cell count of 14.3x10^3^ cells/L, the results of the initial blood workup, which included a thorough metabolic panel, complete blood cell count, erythrocyte sedimentation rate (ESR), and procalcitonin level, were all within normal limits. The tests for blood cultures, Fungitell, and COVID-19 were all negative. Antibodies to syphilis, HIV 1/2, P24 antigen, and toxoplasma IgG and IgM antibodies were all found to be negative in serum samples. Cannabinoids were the only drugs detected in the urine drug test.

CSF was colorless, with a slightly elevated red blood cell count of 63/uL, a normal leukocyte count, and normal total protein and glucose levels. Serological tests for syphilis (via the Venereal Disease Research Laboratory test), Epstein-Barr virus, varicella-zoster virus, cryptococcal antigen, and Lyme disease were all negative. Bacterial and fungal infections were not detected in CSF cultures. The oligoclonal band testing of a CSF sample for autoimmune diseases revealed three well-defined gamma restriction bands that were not present in the patient's corresponding serum sample, indicating abnormal gammaglobulin synthesis in the CNS.

**Figure 1:**
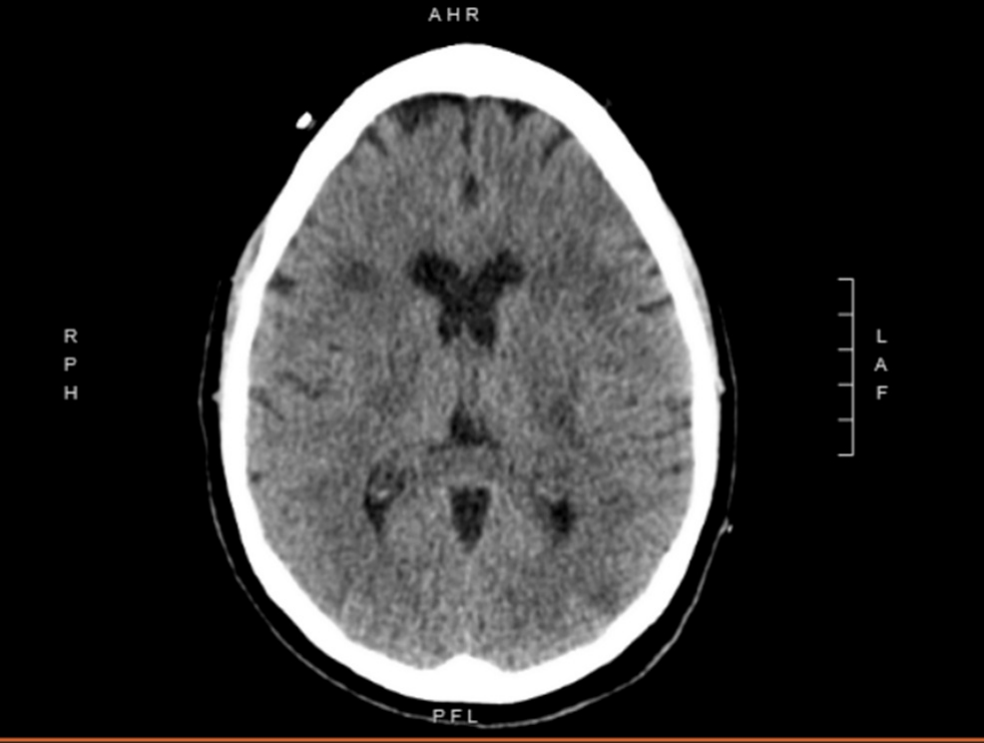
CT scan showing confluent multifocal white matter and basal ganglia hypoattenuation lesions. CT, computed tomography

Initially, computed tomography (CT) revealed multifocal white matter and basal ganglia hypoattenuation lesions that appeared to be confluent (Figure 1). Small vessel ischemic sequela was improbable based on the patient's known medical history. There was no evidence of intracranial hemorrhage or a mass effect.

**Figure 2:**
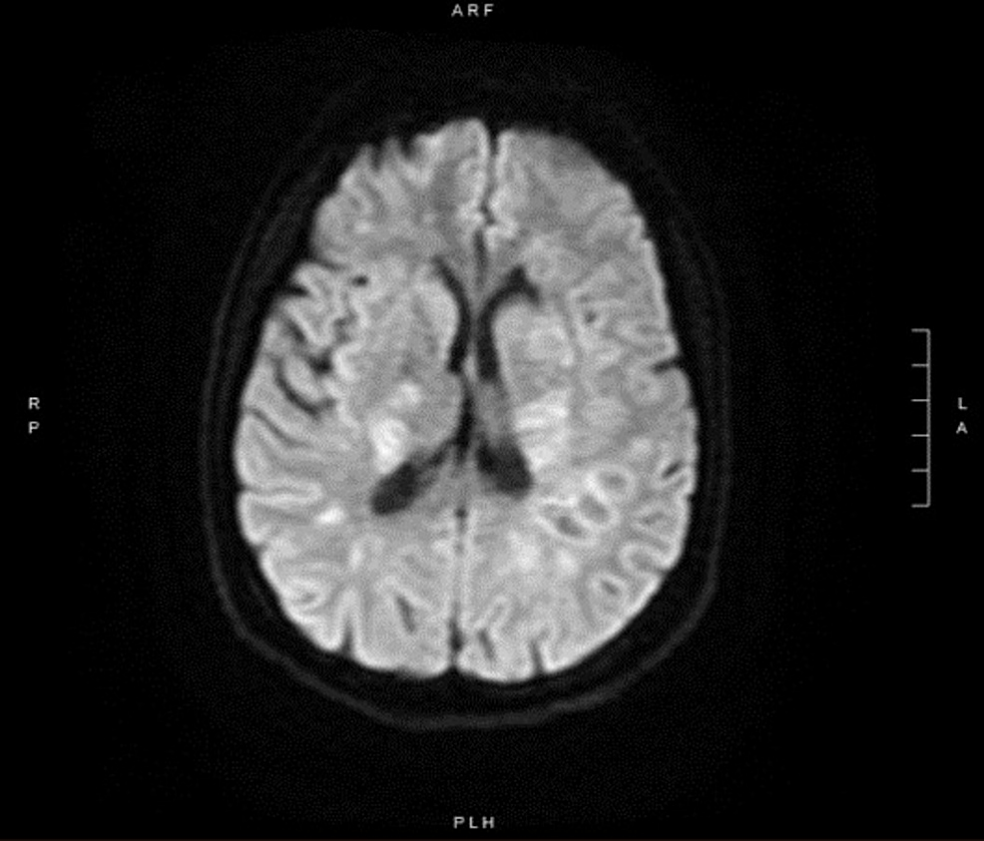
MRI-DWI showing restricted diffusion of innumerable lesions in both central hemispheres. MRI, magnetic resonance imaging; DWI, diffusion-weighted imaging

**Figure 3:**
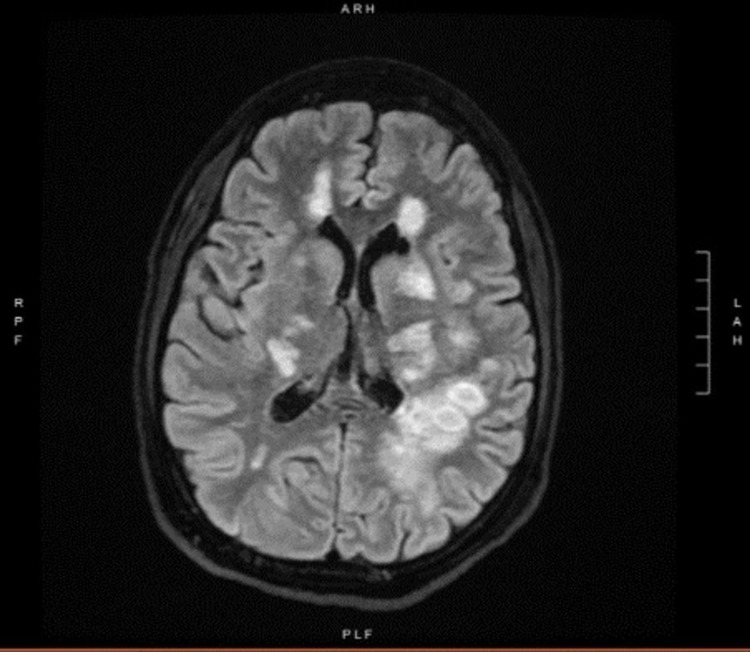
MRI T2-weighted image showing multiple ring-enhancing lesions. MRI, magnetic resonance imaging

A contrast-enhanced magnetic resonance imaging (MRI) of the brain was performed. Diffusion-weighted imaging (DWI) revealed that numerous ring lesions in the cerebrum and cerebellum had restricted diffusion (Figure 2). Multiple T2 hyperintense lesions are seen throughout deep and subcortical white matter of the cerebellum and cerebrum in the T2 and FLAIR (fluid-attenuated inversion recovery) series. Lesions were found in the central medulla, left midbrain, and right midbrain, all of which were confluent with the mid and posterior parietal lobes (Figure 3).

Despite the fact that the patient's blood, serum, and CSF cultures were negative, she was initially treated for a possible brain abscess with empiric antibiotics including vancomycin, ceftriaxone, and metronidazole. Transthoracic and transesophageal echocardiograms were performed, but no valvular vegetations were found.

Testing with an electroencephalogram (EEG) revealed moderate-to-severe generalized slowing. There was no asymmetry or focal findings, and no waveforms or patterns that would indicate a specific etiology for the patient's encephalopathy.

**Figure 4:**
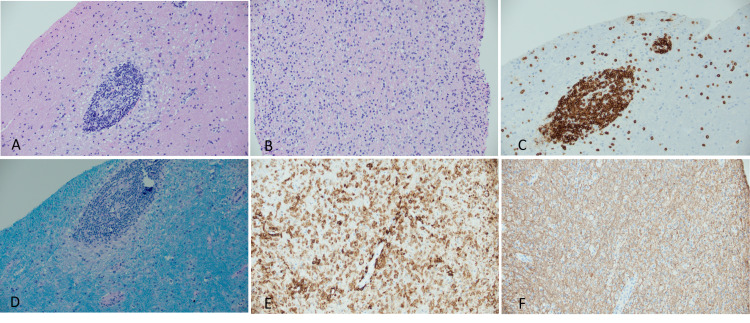
Brain biopsy. (A) White matter with dense perivenular inflammatory cuffs, composed of small lymphocytes, H&E, 20x. (B) White matter with “macrophage-rich lesion” corresponding to the area of active demyelination, H&E, 20x. (C) Perivascular inflammation is composed predominantly of T-lymphocytes, CD3 immunostain, 20x. (D) White matter surrounding areas of perivascular inflammation with collaret of incipient pallor/demyelination, Luxol fast blue stain, 20x. (E) Dense macrophage infiltrates in demyelinating plaque, CD163 immunostain, 20x. (F) Axonal network is overall well preserved, consistent with a demyelinating lesion, neurofilament protein immunostain, 20x.

MRI-guided stereotactic brain biopsy of the left frontal lobe was performed because the patient tested negative for HIV, and her brain MRI with contrast revealed multiple ring-enhancing lesions. Histopathological examination of the brain biopsy revealed white matter with dense perivenular inflammatory cuffs (Figure 4A), white matter with "macrophage-rich lesion" corresponding to the area of active demyelination (Figure 4B), perivascular inflammation composed primarily of T-lymphocytes (Figure 4C), white matter surrounding areas of perivascular inflammation with collaret of incipient pallor/demyelination (Figure 4D), dense macrophage infiltrates in demyelinating plaque (Figure 4E), and axonal network is overall well preserved, consistent with a demyelinating lesion (Figure 4F). All of these findings strongly suggested ADEM.

The patient was given 1 g of methylprednisolone daily for five days, with 10 sessions of plasmapheresis. She made gradual progress over the next few days in resolving her motor deficit and improving her speech deficit. The indexed patient was referred to treatment and recovery services for polysubstance abuse at the time of discharge. The intensity of brain lesions appears to be significantly less visible on follow-up imaging using MRI brain with contrast compared to the patient's initial assessment. On the FLAIR sequence, the signal intensity was greatly improved, as was the area surrounding the suspected perilesional edema. There were no new lesions identified.

## Discussion

ADEM is a rare CNS disorder characterized by widespread demyelination that primarily affects the white matter of the brain and spinal cord. Patients usually present with acute focal or multifocal neurologic symptoms such as encephalopathy, motor, sensory, and oculomotor deficits, as well as dysarthria and encephalopathy [1-3]. The presence of encephalopathy is one of the essential clinical criteria for a diagnosis of ADEM in children, who have the disease at a higher rate than adults. The occurrence of encephalopathy in adults is clinically significant because it aids in the identification of those who are less likely to have another disease, such as multiple sclerosis, and more likely to have ADEM. However, because encephalopathy has not always been a needed criterion for diagnosis, only 20% to 56% of adult cases have been documented [2].

Although the incidence of ADEM is higher in children, it can occur in adults as well, with a reported mortality rate of about 20% [4]. ADEM typically has a monophasic course, but recurrent or multiphasic forms have been reported [5]. CSF analysis in patients with ADEM sometimes reveals changes indicative of an autoimmune disorder such as an oligoclonal band of IgG production. Non-specific EEG abnormalities exist, but due to their low sensitivity and specificity, EEG studies are not routinely required to diagnose ADEM. Because multifocal lesions are present in the subcortical white matter, neuroimaging is a very useful tool in the diagnosis of ADEM [3]. The radiological hallmark of this condition is bilateral and asymmetrical white matter brain lesions on MRI, which are hyperintense on T2-FLAIR sequences [6].

The pathophysiology of ADEM is unknown, but it is thought to be an immunologically mediated demyelinating disorder triggered by a febrile illness or recent vaccination. Direct immune response against cross-reacting antigens (i.e., molecular mimicry) or direct inflammatory damage to myelinated neurons during infection resolution has been proposed as a possible mechanism [7].

There are no randomized controlled trials to determine the most effective treatment regimens for ADEM; however, due to the similar pathogenesis of ADEM and multiple sclerosis, the mainstay treatment for ADEM remains immunosuppression with high-dose intravenous methylprednisolone, plasma exchange, and intravenous immunoglobulin [8].

Our patient had clinical symptoms, a physical examination, and radiological imaging suggestive of ADEM, as well as a CSF profile and histopathology suggestive of ADEM. The patient's urine toxicology was positive for cannabinoids, but due to the patient's altered mental status at the time of presentation, we were unable to obtain more information about cannabis use. However, the patient later revealed a history of cannabis abuse. There had been no recent febrile illness or vaccinations. To the best of our knowledge, there have been no reports of ADEM caused by chronic cannabis abuse, though Samra et al. reported ADEM in a healthy 25-year-old man after using a synthetic cannabinoid called Black Mamba [9]. Other cases of neurological diseases associated with synthetic cannabinoid use have also been reported, though the causative mechanism in those patients was unknown [10]. However, a growing body of research suggests that chronic cannabinoid exposure, particularly during adolescence, can have neurotoxic effects on developing white matter, resulting in decreased white matter coherence, though the physiopathology is unknown. These effects appear in response to long-term cannabis use, implying a link between cannabis abuse and white matter changes [11-12], though more research is needed to confirm this association.

## Conclusions

We reported a challenging case of ADEM associated with chronic cannabis abuse, a clinical entity that should be kept in mind while handling patients with similar presentation. Further studies are needed to better understand the association of neurological manifestations with cannabis abuse. Continuing reporting of anecdotal experiences in managing similar complex scenarios of substance abuse is essential and remains the only reference for clinicians diagnosing and treating this condition.
